# A phase II study of carboplatin and etoposide plus durvalumab for previously untreated extensive-stage small-cell lung cancer (ES-SCLC) patients with a poor performance status (PS): NEJ045A study protocol

**DOI:** 10.1186/s12885-022-10222-1

**Published:** 2022-11-04

**Authors:** Tetsuhiko Asao, Satoshi Watanabe, Takahiro Tanaka, Satoshi Morita, Kunihiko Kobayashi

**Affiliations:** 1 Department of Respiratory Medicine, Juntendo University Graduate School of Medicine, Tokyo, Japan; 2 Department of Respiratory Medicine and Infectious Diseases, Niigata University Graduate School of Medical and Dental Sciences, 1-757 Asahimachidori, Chuouku, Niigata, 951-8510 Japan; 3 Clinical and Translational Research Center, Niigata University Medical and Dental Hospital, Niigata, Japan; 4 Department of Biomedical Statistics and Bioinformatics, Kyoto University Graduate School of Medicine, Kyoto, Japan; 5 Department of Respiratory Medicine, Saitama Medical University International Medical Center, Saitama, Japan

**Keywords:** Small-cell lung cancer, Poor performance status, Immune checkpoint inhibitor, Anti-PD-L1, Durvalumab

## Abstract

**Background:**

Small-cell lung cancer (SCLC) accounts for 12–15% of lung cancers and has a limited prognosis, with approximately one-third of SCLC patients having a poor performance status (PS). Patients with extensive-stage (ES) SCLC and a poor PS have a poor prognosis. For this population, overall survival from carboplatin and etoposide treatment is 7–8 months, and treatment development is an unmet medical need. Recently, the combination of an anti-PD-L1 (a ligand for programmed cell death 1) antibody and platinum-based chemotherapy has become the standard of care for ES-SCLC patients with a good PS (PS 0–1). We hypothesized that the combination of the anti-PD-L1 antibody durvalumab with carboplatin and etoposide would be feasible and effective for such patients.

**Methods:**

We initiated a multicenter phase II study of durvalumab combined with carboplatin and etoposide in previously untreated ES-SCLC patients with a poor PS (PS 2–3). Eligible patients will receive durvalumab plus carboplatin and etoposide every 3 to 4 weeks for up to 4 cycles, followed by durvalumab every 4 weeks until progression or unacceptable toxicity. The dosages of carboplatin and etoposide for the second and subsequent cycles will be adaptively determined based on the adverse events of the first cycle. A total of 56 patients (43 patients with a PS of 2 and 13 patients with a PS of 3) will be enrolled in this study, with a 24-month enrollment period and a 12-month follow-up. The primary endpoint is the tolerability of carboplatin and etoposide plus durvalumab in previously untreated ES-SCLC patients with a poor PS. The secondary endpoints are the 1-year survival rate, objective response rate, progression-free survival, overall survival, ratio of PS improvement, and safety.

**Discussion:**

The results of this study are intended to establish the safety and efficacy of carboplatin and etoposide plus durvalumab in patients with ES-SCLC and a poor PS.

**Trial registration:**

Japan Registry of Clinical Trials (jRCT), jRCTs031200319. Registered 21 January 2021, https://jrct.niph.go.jp/en-latest-detail/jRCTs031200319

## Background

Lung cancer is the leading cause of cancer-related deaths worldwide [1]. Small-cell lung cancer (SCLC) accounts for 12–15% of lung cancers. The age-adjusted incidence rate of SCLC in the U.S. was reported to be approximately 6 per 100,000 in 2016 according to an analysis of the SEER database [2]. SCLC has a limited prognosis (lung cancer-specific 2-year survival rates are only 11% for men and 17% for women), and the treatment of SCLC remains an important clinical challenge [2].

The standard of care for the first-line treatment of extensive-stage (ES) SCLC was platinum-based (platinum-doublet) chemotherapy (cisplatin or carboplatin and etoposide or irinotecan) for many years until the advent of combination therapy with immune checkpoint inhibitors (ICIs). Despite the high objective response rate (ORR) of cisplatin and irinotecan or cisplatin and etoposide (60–80%), the overall survival (OS) duration is approximately only 10–13 months for patients with a good Eastern Cooperative Oncology Group (ECOG) performance status (PS) [3–6].

Because of the rapid progression of SCLC, many patients are in a poor general condition at the time of diagnosis. A previous study reported that 51 (35%) of 145 patients with SCLC had a PS of 2–3 [7]. For patients with a poor PS (PS 2–3), carboplatin and etoposide have been one of the standard-of-care treatments for untreated ES-SCLC based on the Japan Clinical Oncology Group (JCOG) 9702 study [8]. This randomized phase 3 trial compared carboplatin (area under the curve [AUC] 5, day 1) and etoposide (80 mg/m^2^, days 1–3) with cisplatin and etoposide in divided doses (split PE) in patients with a PS of 3 and aged less than 70 years or those with a PS of 0 to 2 and aged 70 years or older. The median progression-free survival (PFS), median OS, and 1-year survival rates of carboplatin and etoposide vs. split PE were 5.2 vs. 4.7 months, 10.6 vs. 9.9 months, and 41% vs. 35%, respectively. In the subgroup analysis, the median OS for patients with a PS of 2–3 treated with carboplatin plus etoposide vs. split PE was 8.3 months vs. 8.1 months. Although grade 3 or 4 hematologic toxicities, such as leukopenia (grade 3 or higher: 54%), neutropenia (95%), anemia (29%), and thrombocytopenia (56%), were observed among all patients treated with carboplatin and etoposide, most toxicities were tolerable, and treatment compliance was favorable. Although adverse events in patients with a poor PS were not reported in this study, 74% of patients had a PS of 0–1 and were aged 70 years or older in the JCOG 9702 study, and patients with a poor PS had more frequent adverse events than those with a good PS.

Recently, the combination of an ICI and platinum-based chemotherapy has become a standard of care for chemotherapy-naïve ES-SCLC patients with a PS of 0–1. Programmed cell death-1 (PD-1) and PD-1 ligand-1 (PD-L1) play an important role in tumor immune evasion. The addition of an anti-PD-L1 antibody to conventional chemotherapy enhances tumor-specific T cell immunity by inhibiting the PD-1/PD-L1 pathway [9]. In a phase III trial (CASPIAN) that compared durvalumab (1500 mg) plus platinum-based chemotherapy with conventional platinum-based chemotherapy (cisplatin 75–80 mg/m^2^ and etoposide 80–100 mg/m^2^ or carboplatin AUC 5–6 and etoposide 80–100 mg/m^2^) in previously untreated ES-SCLC patients, platinum-based chemotherapy and durvalumab significantly prolonged OS, which was the primary endpoint (median OS 13.0 months in the study group vs. 10.3 months in the control group; hazard ratio (HR) 0.73 [95% confidence interval (CI) 0.59–0.91]; *p* = 0.0047) [10]. The response rate was significantly higher in the study treatment group (68%) than in the standard treatment group (58%) (odds ratio 1.56 [95% CI 1.10–2.22]). In a phase III study (IMpower133) on the addition of atezolizumab (1200 mg), an anti-PD-L1 antibody, to conventional platinum-based chemotherapy (carboplatin AUC 5 and etoposide 100 mg/m^2^), carboplatin and etoposide with atezolizumab prolonged OS compared with carboplatin plus etoposide (12.3 months in the study group vs. 10.3 months in the control group; HR 0.70 [95% CI 0.54–0.91]; *p* = 0.007) [11].

The combination of immune checkpoint inhibitors with cytotoxic chemotherapy has a synergistic effect and improves efficacy in ES-SCLC patients with a good PS. However, despite the high frequency of ES-SCLC patients with a poor PS, there remains an unmet need to explore the combination therapy of an ICI and platinum-doublet chemotherapy in these patients. Since SCLC is highly sensitive to cytotoxic chemotherapy, we hypothesized that patients with ES-SCLC and a poor PS may benefit from anti-PD-L1 antibody therapy because tumor shrinkage by anticancer agents is expected to improve PS and tumor immune responses. Therefore, we planned a phase II study to evaluate the safety and efficacy of carboplatin and etoposide plus durvalumab for patients with chemotherapy-naïve ES-SCLC and a poor PS (PS 2–3).

## Methods

### Study design and objectives

**Fig. 1:**
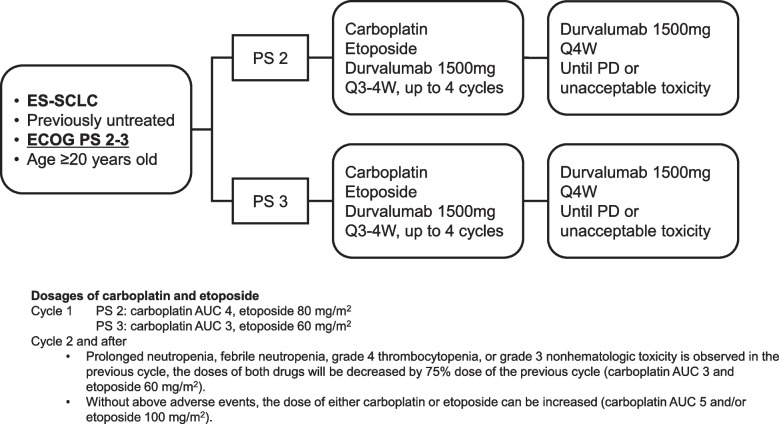
NEJ045A Study Design. Abbreviations: ES-SCLC, extensive-stage small-cell lung cancer; ECOG, Eastern Cooperative Oncology Group; PS, performance status; AUC, area under the curve; PD, progressive disease

NEJ045A is a phase II, nonrandomized, open-label, single-arm trial (Japan Registry of Clinical Trials protocol identification no. jRCTs031200319). The study scheme is shown in Fig. 1. The primary endpoint is the tolerability of carboplatin and etoposide plus durvalumab in previously untreated ES-SCLC patients with a poor PS (PS 2–3). The secondary endpoints are the 1-year survival rate, ORR, PFS, OS, ratio of PS improvement, and safety. Patients will receive carboplatin and etoposide plus 1500 mg durvalumab (day 1) every 3 to 4 weeks in the induction phase, followed by 1500 mg durvalumab every 4 weeks in the maintenance phase until progression or unacceptable toxicity. The initial doses of carboplatin and etoposide will be modified according to PS. In patients with a PS of 2, at the first cycle, patients will receive carboplatin AUC 4 (day 1) and etoposide 80 mg/m^2^ (days 1–3). If prolonged neutropenia (grade 4 or higher for 4 days or more), febrile neutropenia, grade 4 or higher thrombocytopenia, or grade 3 or higher nonhematologic toxicity is observed in the previous cycle, the doses of both drugs will be decreased by 75% those of the previous cycle: namely, carboplatin AUC 3 and etoposide 60 mg/m^2^. If the above adverse events are not observed in the previous cycle, the dose of either carboplatin or etoposide can be increased (carboplatin AUC 5 and/or etoposide 100 mg/m^2^), or the same dose can be continued. The dose of durvalumab will be fixed at 1500 mg. To ensure safety, six patients with a PS of 2 will be enrolled in the first study and then evaluated by the efficacy and safety evaluation committee. In patients with a PS of 3, at the first cycle, patients will receive carboplatin AUC 4 (day 1) and etoposide 80 mg/m^2^ (days 1–3). If the above adverse events are not observed in the previous cycle, the dose of either carboplatin or etoposide can be increased, or the same dose can be continued. The use of the granulocyte colony-stimulating factor is allowed as the primary and secondary prophylaxis of febrile neutropenia.

### Key eligibility criteria

Key inclusion and exclusion criteria are shown in Table 1.

**Table 1:** Key patient eligibility criteria

Inclusion Criteria	
• Histologically or cytologically confirmed SCLC • ES-SCLC that is not amenable to surgery or curative irradiation • No prior chemotherapy for SCLC • ECOG PS of 2 or 3 • Age 20 years or older at the time of consent • Patients with measurable lesions that are target lesions according to the RECIST version 1.1 • Adequate organ function • Neutrophil count: 1500/mm^3^ or more • Hemoglobin: 9.0 g/dL or more • Platelet count: 10.0 × 10^4^/mm^3^ or more • AST: 2.5 times or less the upper limit of the institutional standard (5 times or less the upper limit of the institutional standard in the case of liver metastasis) • ALT: 2.5 times or less the upper limit of the institutional reference value (5 times or less the upper limit of the institutional reference value if liver metastases are present) • Total bilirubin: 1.5 times or less the upper limit of the institutional standard (3 times or less in the case of indirect hyperbilirubinemia such as Gilbert’s syndrome) • SpO_2_ 93% or higher (or PaO_2_ 70 Torr or higher) • Serum creatinine: less than 1.5 mg/dL (or creatinine clearance 45 mL/min or more) • Written informed consent	
Exclusion Criteria	
• Patients with a usual interstitial pneumonia (UIP) pattern or probable UIP pattern on chest CT • Patients with meningeal carcinomatosis • Patients with a history of radiation to the primary lesion • Patients with pleural effusion, pericardial effusion, or ascites requiring drainage • Patients with coexisting autoimmune diseases (myasthenia gravis, myositis, autoimmune hepatitis, systemic lupus erythematosus, rheumatoid arthritis, inflammatory bowel disease, vasculitis, Sjogren’s syndrome, multiple sclerosis, etc.) • Patients with serious complications (active infection, symptomatic congestive heart failure, uncontrolled hypertension, unstable angina pectoris, fatal arrhythmia, respiratory, hepatic or renal disease, etc.) • Patients with concurrent multiple cancers or heterogeneous multiple cancers with a disease-free interval of 5 years or less • Other patients who are judged by the principal investigator or subinvestigator to be inappropriate for participation in this study	

*Abbreviations*: *ES-SCLC* Extensive-stage small-cell lung cancer, *ECOG* Eastern Cooperative Oncology Group, *PS* Performance status, *RECIST* Response Evaluation Criteria in Solid Tumors, *AST* Aspartate aminotransferase, *ALT* Alanine aminotransferase, *SpO*_*2*_ Percutaneous oxygen saturation, *PaO*_*2*_ Partial pressure of arterial oxygen, *CT* Computed tomography

### Statistical analysis

The primary endpoint, the tolerability of carboplatin and etoposide plus durvalumab in untreated ES-SCLC patients with a poor PS, will be evaluated based on the percentage of patients who complete four cycles of carboplatin and etoposide plus durvalumab. In the JCOG 9702 study, which evaluated carboplatin and etoposide in ES-SCLC patients with a PS of 3 and aged less than 70 years or with a PS of 0–2 and aged 70 years or more, demonstrated that 63% of patients completed 4 cycles [8]. Our study will include only patients with a poor PS; therefore, if 50% of patients complete 4 cycles, the study treatment will be considered tolerable. If the expected percentage of completion in patients with a PS of 2 is 50%, the threshold percentage of completion is 33%, the one-sided alpha error is 0.1, and the beta error is 0.2, the required number of patients is calculated to be 41 based on the exact binomial distribution. If the expected percentage of completion in patients with a PS of 3 is 50%, the threshold percentage of completion is 20%, the one-sided alpha error is 0.1, and the beta error is 0.2, the required number of patients is calculated to be 12 based on the exact binomial distribution.

The total number of patients was calculated to be 56, considering the patients excluded from the analysis due to censoring. For the 1-year survival rate, which is the most important secondary endpoint, under the expected value of 26%, a threshold of 13%, and alpha error of 0.1 (one-sided), the power of the exact test for the binomial ratio is calculated to be 85 and 78% under all 53 patients (sum of those with a PS of 2 and 3) and 41 patients with a PS of 2, respectively.

This study was approved by the Niigata University Central Review Board of Clinical Research. All procedures will be performed in accordance with the ethical standards of the institutional and/or national research committee and with the 2013 Declaration of Helsinki and its later amendments or comparable ethical standards. All patients must provide written informed consent.

### Participating institutions

Participating institutions are 31 hospitals throughout Japan that are members of the North East Japan Study Group (NEJSG).

## Discussion

The safety and efficacy of adding ICIs to platinum-doublet chemotherapy for ES-SCLC patients with a poor PS are unclear, and this is the significance of this study. A systematic review demonstrated that non-small cell lung cancer patients with a poor PS had a worse prognosis than those with a good PS [12]. Furthermore, a heavy tumor burden was associated with poor responsiveness to ICIs [13] Because platinum-doublet chemotherapy has a high response rate in ES-SCLC patients, a decrease in the tumor volume by cytotoxic chemotherapy results in improved PS, antitumor immunity and responsiveness to ICIs. To ensure safety, the initial chemotherapy dose will be started at a reduced level, but the design allows for dose escalation from the second cycle onward so that the effect on efficacy is minimized.

## Conclusion

This is the first trial to examine the safety and efficacy of carboplatin and etoposide plus durvalumab for patients with ES-SCLC and a poor PS (PS 2–3). This study will provide new insight into the utilization of immune checkpoint inhibitors in these subjects.

## Data Availability

The datasets used and/or analyzed during the current study are available from the corresponding author upon reasonable request.

## Abbreviations

AUC: Area under the curve
CI: Confidence interval
ECOG: Eastern Cooperative Oncology Group
ES: Extensive stage
HR: Hazard ratio
ICI: Immune checkpoint inhibitor
JCOG: Japan Clinical Oncology Group
jRCT: Japan Registry of Clinical Trials
NEJSG: North East Japan Study Group
NSCLC: Non-small-cell lung cancer
ORR: Objective response rate
OS: Overall survival
PD-1: Programmed cell death-1
PD-L1: Programmed cell death ligand-1
PFS: Progression-free survival
PE: Cisplatin and etoposide
PS: Performance status
SCLC: Small-cell lung cancer
